# Accuracy of static computer-assisted implant placement in anterior and posterior sites by clinicians new to implant dentistry: in vitro comparison of fully guided, pilot-guided, and freehand protocols

**DOI:** 10.1186/s40729-020-0205-3

**Published:** 2020-03-11

**Authors:** Jaafar Abduo, Douglas Lau

**Affiliations:** 1 Associate Professor in Prosthodontics, Convenor of Postgraduate Diploma in Clinical Dentistry (Implants), Melbourne Dental School, Melbourne University, 720 Swanston Street, Melbourne, VIC 3010 Australia; 2 Periodontist, Private Practice, Melbourne University, Melbourne, VIC Australia

**Keywords:** 3D, CAD/CAM, Computer-guided surgery, Single implant, Surgical guides

## Abstract

**Background:**

One of the challenges encountered by clinicians new to implant dentistry is the determination and controlling of implant location. This study compared the accuracy of fully guided (FG) and pilot-guided (PG) static computer-assisted implant placement (sCAIP) protocols against the conventional freehand (FH) protocol for placing single anterior and posterior implants by recently introduced clinicians to implant dentistry.

**Material and methods:**

Ten clinicians new to implant dentistry inserted one anterior (central incisor) and one posterior (first molar) implants per protocol in training maxillary models. The FG protocol involved drilling and implant placement through the guide, while the PG protocol controlled the pilot drilling only. The FH implant placement was completed without the aid of any guide. A total of 30 models were used, and 60 implants were inserted. The implant vertical, horizontal neck, horizontal apex, and angle deviations from planned positions were calculated.

**Results:**

The FG protocol provided the most accurate implant placement in relation to horizontal neck (0.47 mm–0.52 mm), horizontal apex (0.71 mm–0.74 mm), and angle deviations (2.42^o^–2.61^o^). The vertical deviation was not significantly different among the different protocols. The PG protocol was generally similar to the FH protocol with a horizontal neck deviation of 1.01 mm–1.14 mm, horizontal apex deviation of 1.02 mm–1.35 mm, and angle deviation of 4.65^o^–7.79^o^. The FG protocol showed similarity in the accuracy of the anterior and posterior implants. There was a tendency for inferior accuracy for posterior implants compared with anterior implants for the PG and FH protocols.

**Conclusions:**

In the hands of recently introduced clinicians to implant dentistry, it appears that the accuracy of the FG protocol was superior to the other protocols and was not influenced by the position of the implants. The PG and FH protocols showed inferior accuracy for posterior implants compared with anterior implants.

## Background

Implant treatment is a growing field in dentistry, and many clinicians aim to increase their scope of practice by including such treatment. One of the main challenges encountered by clinicians new to implant dentistry is the determination and controlling of implant location. It is the consensus that implant placement must be planned to achieve an acceptable position for an ideal restorative outcome [1, 2]. Poorly placed implants were demonstrated to be associated with increased marginal bone loss, and may lead to the violation of nearby vital anatomic structures [1–3]. Restoration of poorly placed implants may not have an optimal morphology and emergence profile, which can affect esthetics and impede plaque control. In addition, restoring poorly placed implants is technically far more challenging with increased time and cost [1–3].

While the conventional approach combines the planned restorative outcome with the 3D radiographic information via a radiographic guide, transferring this information to execute the osteotomy at the planned position and angulation is challenging. To overcome this problem, surgical guides were advocated to control implant location, angulation, drilling, and subsequent positioning [4, 5]. Today, with the advancement of digital technologies [6], static computer-assisted implant placement (sCAIP) protocols were proposed as alternatives [7–9]. They involve using commercial software programs to decide on the ideal implant position, digitally designing the surgical guide, and producing the surgical guide by means of 3D printing or milling [10]. Further, manufacturing companies provide surgical guides with prefabricated metal sleeves and drilling handles that correspond to the implant surgical tools, which facilitate exacting implant placement. In addition, it has been proposed that implant placement according to sCAIP protocols are easier, simpler, and more predictable than conventional implant placements [11–13]. Some studies showed that the accuracy of sCAIP implants is less influenced by the lack of experience of the operator [12–14].

Currently, there are two protocols for sCAIP: fully guided (FG) and pilot-guided (PG) protocols [15, 16]. The FG protocol is related to implant manufacturers and has the advantage of controlling all the drilling, tapping, and implant placement through the surgical guide. The PG protocol is an abbreviated form of guided surgery, and only guides the pilot drill. The rest of the surgical procedure is completed freehand. Frequently, the PG protocol is related to open source software programs that allow guide production by 3rd party 3D printers. As a result, the PG protocol is generally more economical than the FG protocol. However, the PG protocol cannot control all the steps of implant placement.

Despite all the advantages of sCAIP protocols, several studies reported that they are still prone to errors and complications [7–9, 17, 18]. The FG and PG protocols still require thorough planning and surgical understanding and skills [11]. For multiple implants and long-span edentulous ridges, guided surgery has the advantages of being more reliable, more comfortable for the patient, and more representative of the restorative planning [11]. However, while applicable for single implant placements, the merit of the new technologies is yet to be established in order to justify their routine use. In addition, the differences between FG and PG protocols have to be determined for single implant placements. Specifically, the influence of the implant location (anterior vs. posterior) on the accuracy of the different protocols will be of relevance to clinicians who are building their experience in implant dentistry. Therefore, this laboratory study aims to compare the accuracy of sCAIP protocols (FG and PG) against the conventional freehand (FH) protocol for single anterior and posterior implant placements by clinicians new to implant dentistry. The null hypotheses are (1) there is no difference in the accuracy of the three protocols, and (2) there is no influence of the location of the implant on the accuracy of placement.

## Methods

A total of 10 qualified clinicians with a minimum of 3 years of general practice experience were invited to participate in the study. The number of participants was similar to previously published studies [12, 19], and was confirmed by sample size calculation. A mean horizontal deviation of 1 mm and an expected standard deviation of 0.75 mm that were reported from earlier studies [13, 19] were used for the calculation. With the assumption of 80% statistical power and a 5% significance level, at least nine clinicians were needed to participate in the study. The clinicians were new to implant dentistry and were undertaking formal implant training at Melbourne Dental School, Melbourne University. As part of their training, they had covered the principles of restorative and surgical implant treatment. Ethics approval was obtained from the University of Melbourne Human Research Ethics Committee (1851406.1).

### Model fabrication

**Fig. 1:**
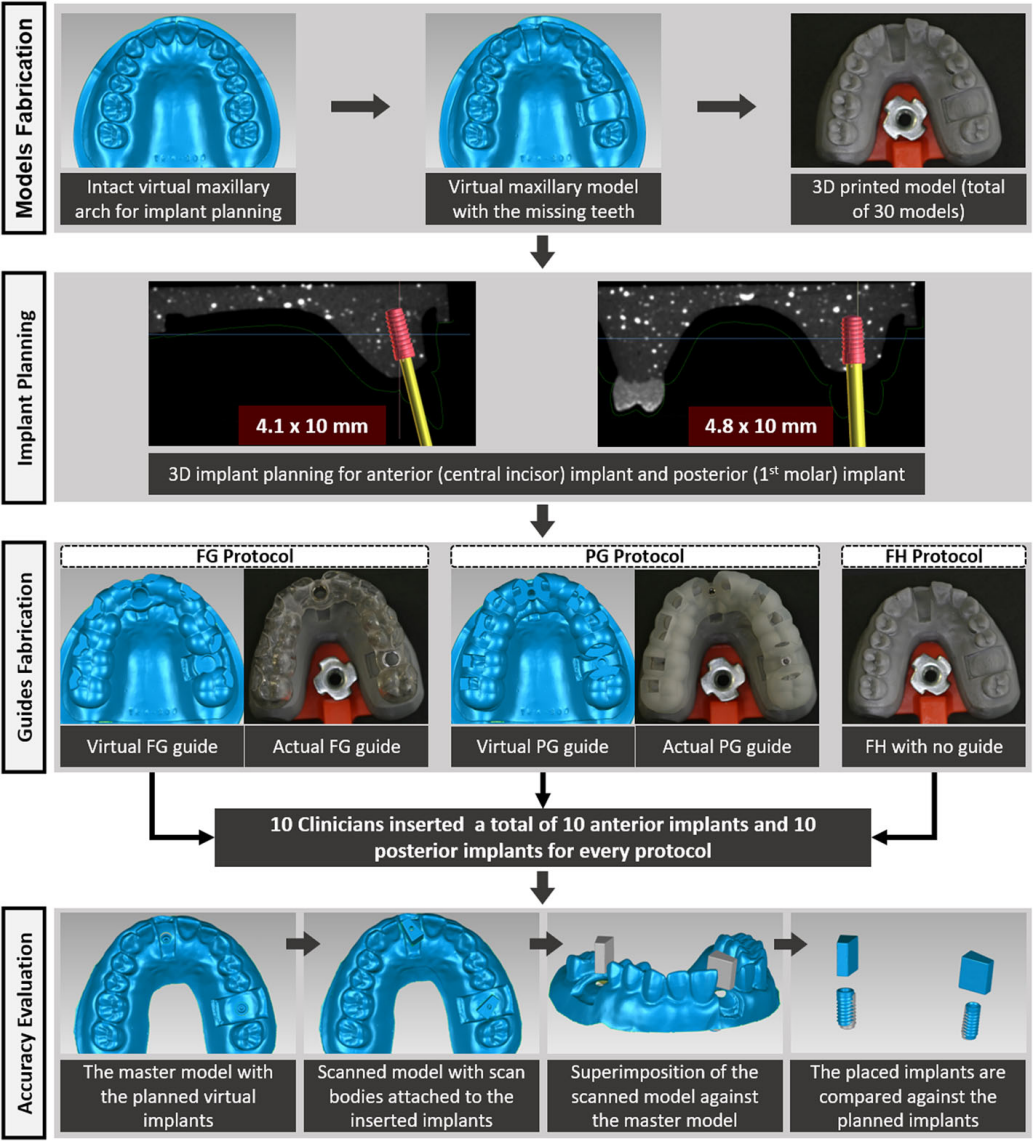
Flowchart summarizing the different phases of the experiment

The different phases of the experiment were summarized in Fig. 1. The maxillary Nissin training model (Nissin Dental Products Inc., Kyoto, Japan) was used to simulate clinical patient presentations. An intact Nissin model was scanned by a laboratory surface scanner (Identica T300, Medit Identica, DT Technologies, Davenport, IA) to generate an ideal virtual maxillary arch for implant planning. The right central incisor, the left first molar, and their associated tissue formers were removed; and the sockets were sealed with wax. Subsequently, the modified model was scanned by the laboratory scanner to produce a virtual model with the missing teeth. The virtual model was converted to a surface tessellation language (STL) format that was used to produce physical polyurethane models by a 3D printer (ProJet, 3510 DP Pro, 3D systems, Rock Hill, SC, USA). For each clinician, 3 polyurethane models were fabricated to allow for implant placement according to the different implant placement protocols. 3D printing was implemented to ensure consistency and similar accuracy of all the produced models. To simulate the clinical scenario, the polyurethane models were fixed on mounting plates compatible with the maxillary compartment of manikin heads. In addition, a standard mandibular Nissin model was attached to the lower compartment of the manikin heads.

The soft tissue silicone former was removed from the Nissin model to simulate bone anatomy. Subsequently, this model was duplicated with clear resin material mixed with barium sulfate and scanned by a cone beam computed tomography (CBCT) machine to generate cross-sectional DICOM images.

### Implant planning and guide fabrication

The DICOM images were imported to the implant planning software programs. For the FH protocol, the 2D DICOM images were visualized on a computer screen to decide on the ideal implant position for the anterior and posterior implants. The clinicians had access to the planning images to allow for ideal FH implant placement. For the FG protocol, the DICOM data were imported to coDiagnostiX software (Dental Wings, Montreal, Canada), which is closely related to the implant company, and can design surgical guides that control all steps of implant surgery. For the PG protocol, the DICOM data were viewed in the Blue Sky Bio software (Grayslake, IL, USA), which is an open-source software that is suitable for designing surgical guides for pilot drilling.

For the FG protocol, the virtual cast and the 3D CBCT image were combined by the software, and the virtual intact model was used to simulate ideal teeth replacement. The virtual intact model provided outline of the planned restorations that dictated the implant position. This was followed by placement of virtual implants within the simulated bone in a favorable 3D position. According to the planned implant position, a surgical guide was designed and produced by a commercial dental laboratory by milling through a 5-axis milling unit (DWX-51D, Roland, Sydney, NSW, Australia). Two Straumann metal sleeves of the FG protocol were attached on every guide. The metal sleeves had a 5-mm diameter and were provided by the implant company to accept all the drilling components and the implants. The PG protocol followed similar steps, and the software was used to design surgical guide with pilot drill holes corresponding to the location of the implants. The STL file of the guide was transferred to a 3D printer (ProJet, 3510 DP Pro, 3D systems, Rock Hill, SC, USA) to produce the surgical guides. After printing the guides, two Straumann pilot drilling sleeves (2.2 mm diameter) were inserted. After the virtual implant planning, an STL file of the virtual model with the planned implants was extracted. This model served as a master model to which all the placed implants were compared to.

### Implant placement

For all the protocols, straight bone level Straumann dummy implants were planned. The anterior implants were 4.1 × 10 mm, while the posterior implants were 4.8 × 10 mm. The anterior implants were planned to be placed 2 mm subcrestal, while the posterior implants were planned to be placed 1 mm subcrestal.

For the conventional protocols, the clinicians had access to physical intact Nissin model casts that represented ideal tooth anatomy and DICOM images with implant planning. For the FG protocol, the steps were provided by the Dental Wings software, that were followed for each implant placement. This involved pilot drilling, sequential drilling, osteotomy profiling and implant placement. The PG protocol only allowed for pilot drilling short of the planned drill depth. The rest of the steps were completed freehand as per the conventional FH protocol.

All the participants inserted the implants according to the conventional protocol first, followed by the PG and FG protocols. This ensured that clinicians did not practice placing the implants with a guide before the FH protocol implant placement.

### Accuracy evaluation

**Fig. 2:**
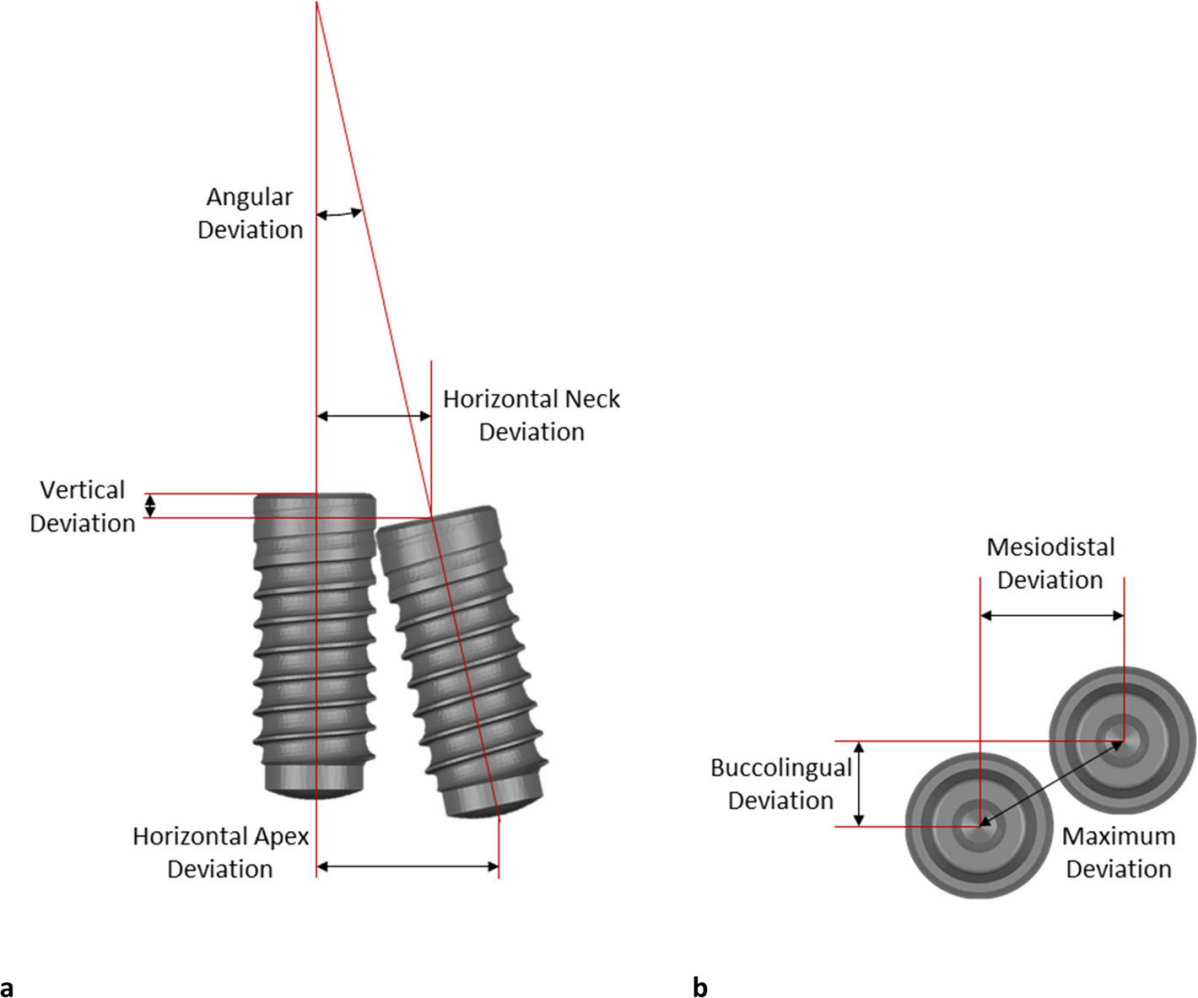
**a** Schematic diagram illustrating the measurement of vertical, horizontal neck, horizontal apex, and angle deviations. **b** Three forms of horizontal deviation were measured: maximum, mesiodistal, and buccolingual directions

For accuracy evaluation of the final implant placement, laboratory scan bodies (ZFX Scan body, ZFX Dental, Zimmer Biomet, Warsaw, IN, USA) were attached to the inserted implants in each polyurethane model. The models with the scan bodies were scanned by a laboratory scanner to generate a virtual model of the cast and implant position. Subsequently, the position of the placed implant was compared against the position of the planned implant at the virtual master model. This was completed by superimposing the final virtual model against the virtual master model by a 3D rendering software (Geomagic Studio, Raindrop, Geomagic Inc., Research Triangle Park, NC, USA). Since the teeth were stable landmarks of all the models, they were used for the superimposition. The superimposition consisted of point-to-point registration followed by automated registration to obtain the best fit between the 2 virtual models. Eventually, each placed implant was spatially related to the planned implant, which allowed for the measurement of the deviation. The deviation of implant position was measured by calculating the following variables: implant vertical deviation, horizontal neck deviation, horizontal apex deviation, and implant angle deviation (Fig. 2).

The vertical deviation was measured by calculating the discrepancy along the long axis of the planned implant at the center of the platform (Fig. 2a). In addition to the magnitude of the deviation, the direction of the error was determined. The horizontal deviations were measured at the neck and the apex of the planned implant. The angle deviation was computed by measuring the angle of the long axis of 2 implants. The direction of the angle deviation was measured by relating the apex location of the placed implant to the planned implant. Maximal, buccolingual, and mesiodistal deviations of the horizontal and angle deviations were determined (Fig. 2b).

### Statistics

For each variable, the mean and standard deviation (SD) were measured. After confirming the normality of the data, the one-way analysis of variance test was applied to determine the statistical difference among the groups. In the case of the presence of a significant difference, the Tukey post hoc test was applied. In addition, for each variable, the difference between the anterior and posterior implants was determined. All the statistical tests were conducted via the SPSS software package (SPSS for Windows, version 23, SPSS Inc., Chicago, IL, USA). The level of significance was set at 0.05. The mesiodistal and buccolingual deviations of each implant of every protocol were blotted in scatter diagrams.

## Results

**Fig. 3:**
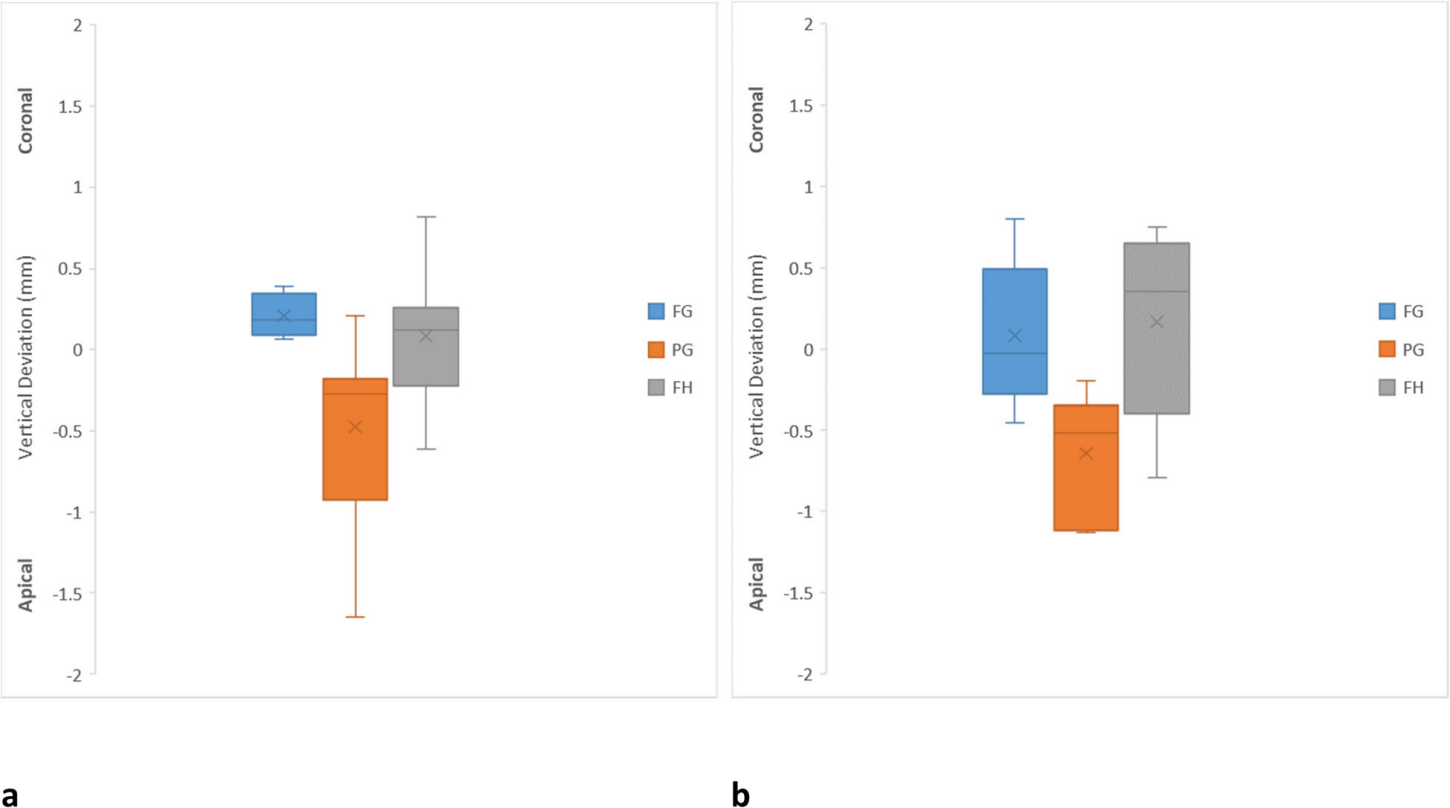
Box plot diagrams illustrating the distribution of vertical deviation of each protocol. **a** Anterior implants. **b** Posterior implants

In general, for all the variables, there was a tendency for the FG protocol to yield more accurate implant placement than other protocols (Table 1). In relation to vertical deviation, the PG protocol seemed to be associated with more errors. However, there was no significant difference in vertical deviation among all the protocols. Figure 3 indicates that the PG protocol was associated with deeper implant placement than the planned implant location for anterior (0.53 ± 0.52 mm) and posterior (0.64 ± 0.37 mm) implants. The FH protocol had less vertical deviation than PG protocol for anterior (0.30 ± 0.24 mm) and posterior (0.49 ± 0.22 mm) implants. The FG protocol had a minimal deviation for the anterior (0.21 ± 0.12 mm) and posterior (0.34 ± 0.23 mm) implants, which tended to be slightly above the planned implants. For all the protocols, the anterior and posterior implants exhibited similar vertical deviations.

**Table 1:** Summary of implant vertical, horizontal and angle deviations from the planned implant

	Vertical implant deviation						
	Anterior implant			Posterior implant			*p* values between anterior and posterior implants
	FG	PG	FH	FG	PG	FH	
Mean (mm)	0.21	0.53	0.30	0.34	0.64	0.49	FG = 0.07
SD (mm)	0.12	0.52	0.24	0.23	0.37	0.22	PG = 0.27
Maximum (mm)	0.39	1.65	0.81	0.80	1.13	0.80	FH = 0.05
Minimum (mm)	0.09	0.05	0.07	0.04	0.20	0.07	
*p* values	All groups = 0.12			All groups = 0.08			
	Maximum horizontal implant neck deviation						
	Anterior implant			Posterior implant			*p* values between anterior and posterior implants
	FG	PG	FH	FG	PG	FH	
Mean (mm)	0.47	1.14	0.79	0.52	1.01	1.27	FG = 0.35
SD (mm)	0.25	0.47	0.26	0.26	0.29	0.22	PG = 0.23
Maximum (mm)	1.07	2.17	1.35	0.99	1.41	1.62	FH = 0.0003
Minimum (mm)	0.16	0.44	0.32	0.37	0.34	0.99	
*p* values	All groups = 0.001			All groups = 0.000			
	FG vs PG = 0.001			FG vs PG = 0.001			
	FG vs FH = 0.13			FG vs FH = 0.000			
	PG vs FH = 0.90			PG vs FH = 0.08			
	Maximum horizontal implant apex deviation						
	Anterior implant			Posterior implant			*p* values between anterior and posterior implants
	FG	PG	FH	FG	PG	FH	
Mean (mm)	0.71	1.02	1.12	0.74	1.35	1.81	FG = 0.37
SD (mm)	0.24	0.54	0.71	0.23	0.55	0.53	PG = 0.10
Maximum (mm)	1.12	1.90	2.30	1.03	2.17	2.47	FH = 0.01
Minimum (mm)	0.27	0.28	0.25	0.44	0.56	1.17	
*p* values	All groups = 0.22			All groups = 0.000			
				FG vs PG = 0.02			
				FG vs FH = 0.00			
				PG vs FH = 0.08			
	Maximum implant angle deviation						
	Anterior implant			Posterior implant			*p* values between anterior and posterior implants
	FG	PG	FH	FG	PG	FH	
Mean (°)	2.42	4.65	4.79	2.61	7.79	4.77	FG = 0.35
SD (°)	0.98	1.78	2.08	1.23	2.64	2.09	PG = 0.003
Maximum (°)	3.91	9.29	7.40	5.07	12.79	8.21	FH = 0.49
Minimum (°)	1.03	2.80	0.89	0.91	4.28	1.26	
*p* values	All groups = 0.01			All groups = 0.000			
	FG vs PG = 0.02			FG vs PG = 0.000			
	FG vs FH = 0.01			FG vs FH = 0.07			
	PG vs FH = 0.98			PG vs FH = 0.01

**Fig. 4:**
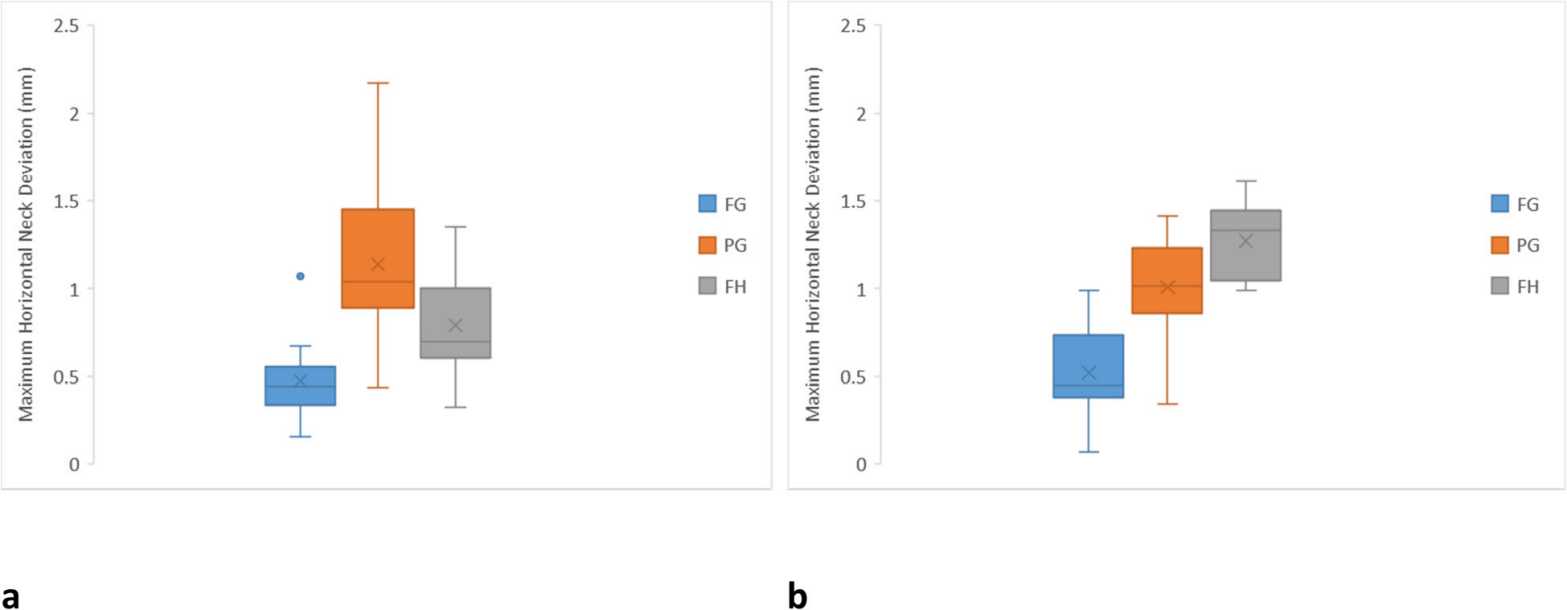
Box plot diagrams illustrating the distribution of maximum horizontal neck deviation of each protocol. **a** Anterior implants. **b** Posterior implants

**Fig. 5:**
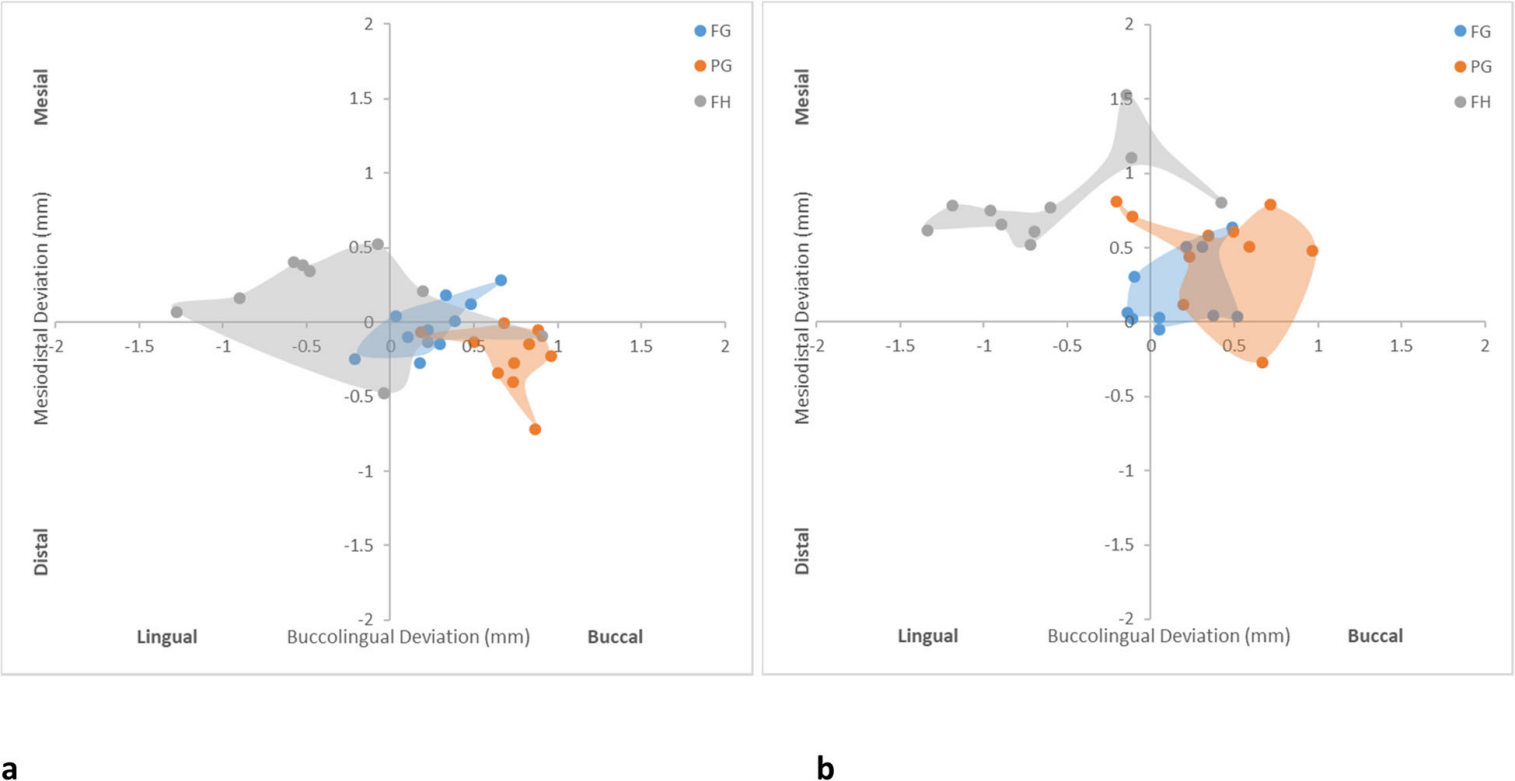
Scatter diagrams illustrating the distribution of horizontal neck deviation of each protocol. **a** Anterior implants. **b** Posterior implants

For the maximum horizontal neck deviation (Fig. 4), the PG protocol was most inferior (1.14 ± 0.47 mm), followed by FH (0.79 ± 0.26 mm) and FG (0.47 ± 0.25 mm) protocols for the anterior implants. For the posterior implants, the FH protocol was most inferior (1.27 ± 0.22 mm), while the FG protocol was most superior (0.52 ± 0.26 mm), followed by the PG protocol (1.01 ± 0.29 mm). The FH and PG protocols were not significantly different in any comparison. The FG and PG protocols seemed less affected by the location of the implant. The FH protocol showed significantly more errors to posterior implants than anterior implants. Figure 5 shows that the FG protocol was associated with implants being centered around 0, indicating the least deviation buccolingually and mesiodistally. The implants of the PG protocol were prominently positioned buccally. The FH protocol appeared to have a wider distribution especially at the buccolingual direction.

**Fig. 6:**
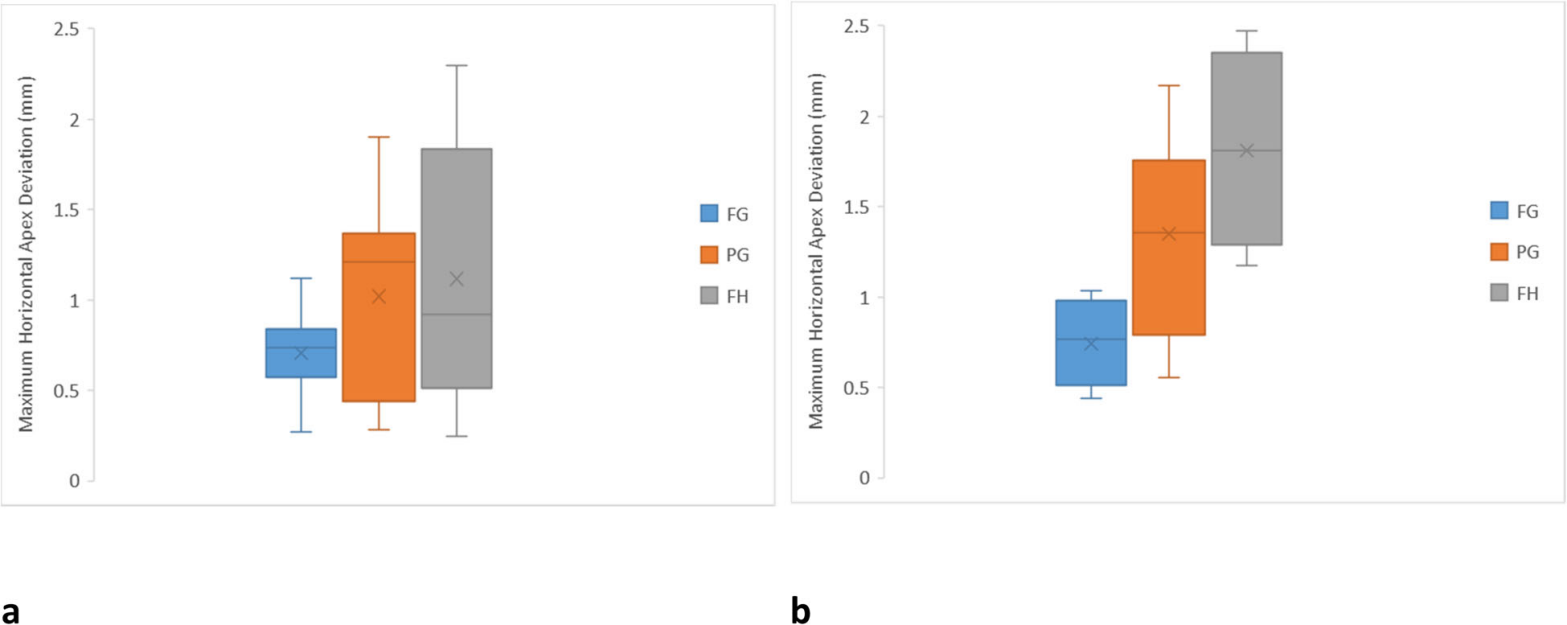
Box plot diagrams illustrating the distribution of maximum horizontal apex deviation of each protocol. **a** Anterior implants. **b** Posterior implants

**Fig. 7:**
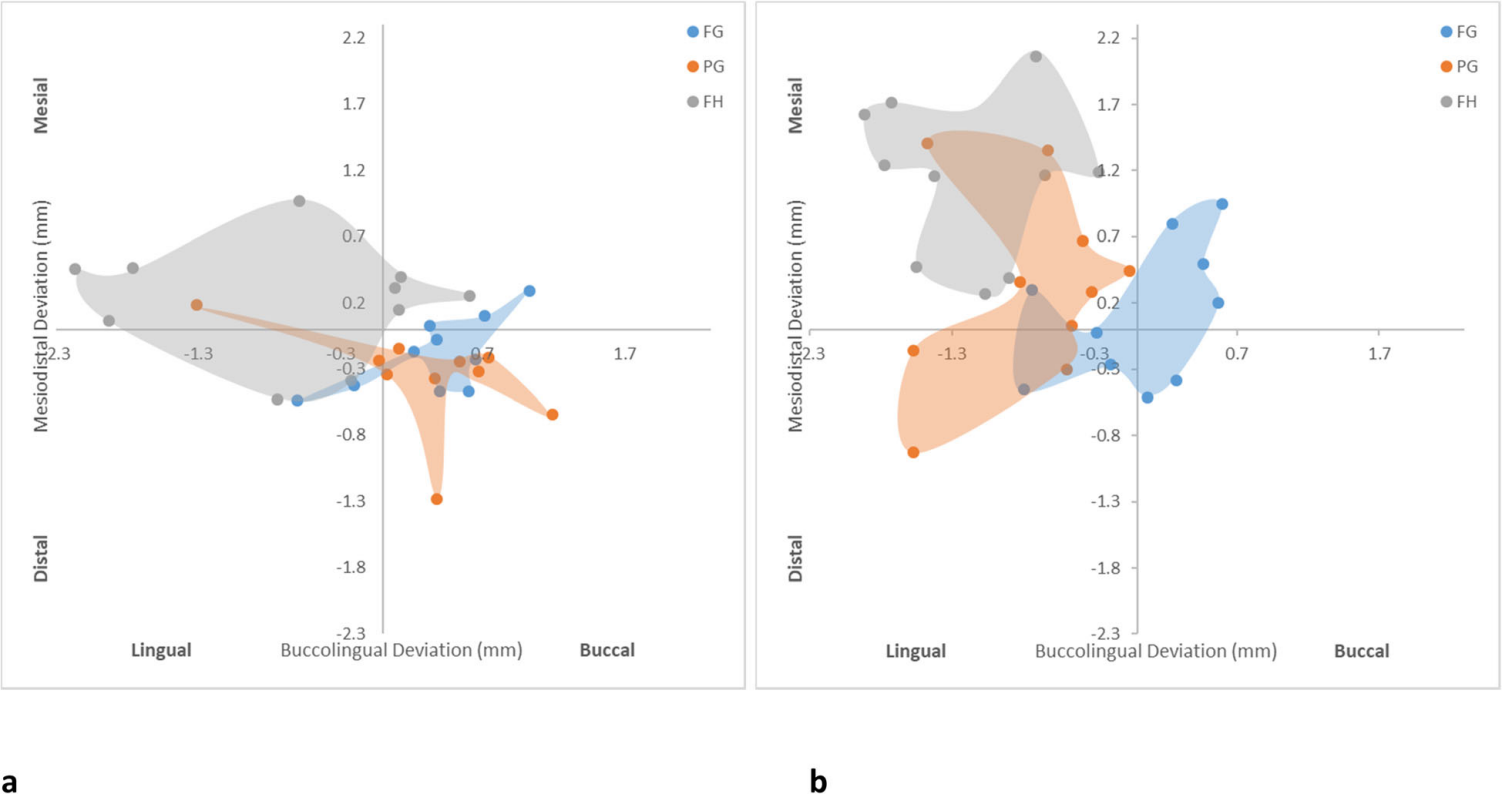
Scatter diagrams illustrating the distribution of horizontal neck deviation of each protocol. **a** Anterior implants. **b** Posterior implants

For the maximum apex deviation (Fig. 6), the FG protocol (0.71 ± 0.24 mm) was slightly more accurate for anterior implants, followed by PG (1.02 ± 0.54 mm) and FH (1.12 ± 0.71 mm) protocols, respectively. However, the difference among the protocols was insignificant. For the posterior implants, there was a clear tendency for the FG protocol (0.74 ± 0.23 mm) to be more accurate, followed by PG (1.35 ± 0.55 mm) and FH (1.81 ± 0.53 mm) protocols, respectively. As per the neck deviation, the apices of the FG and PG protocol implants were less affected by the location, while the FH protocol showed significantly greater errors with posterior implants than anterior implants. Figure 7 confirms the overall accuracy of the FG protocol for anterior and posterior implants in being closer to the center of the graph. For the PG protocol, the anterior implant apices were skewed to the distobuccal aspect, while the posterior implant apices were placed more lingually. The FH protocol anterior implant apices generally exhibited more variation and were skewed more lingually, while the posterior implant apices were predominantly located lingually.

**Fig. 8:**
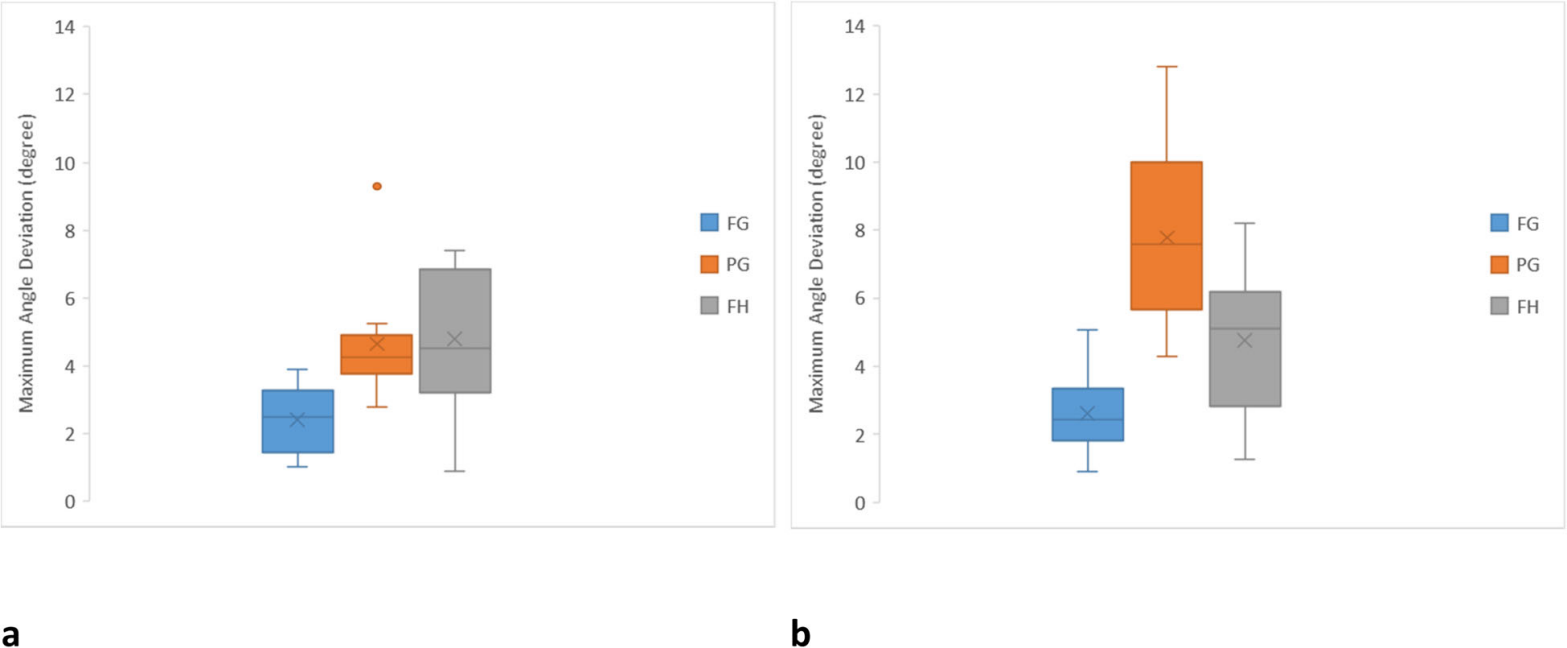
Box plot diagrams illustrating the distribution of maximum angle deviation of each protocol. **a** Anterior implants. **b** Posterior implants

**Fig. 9:**
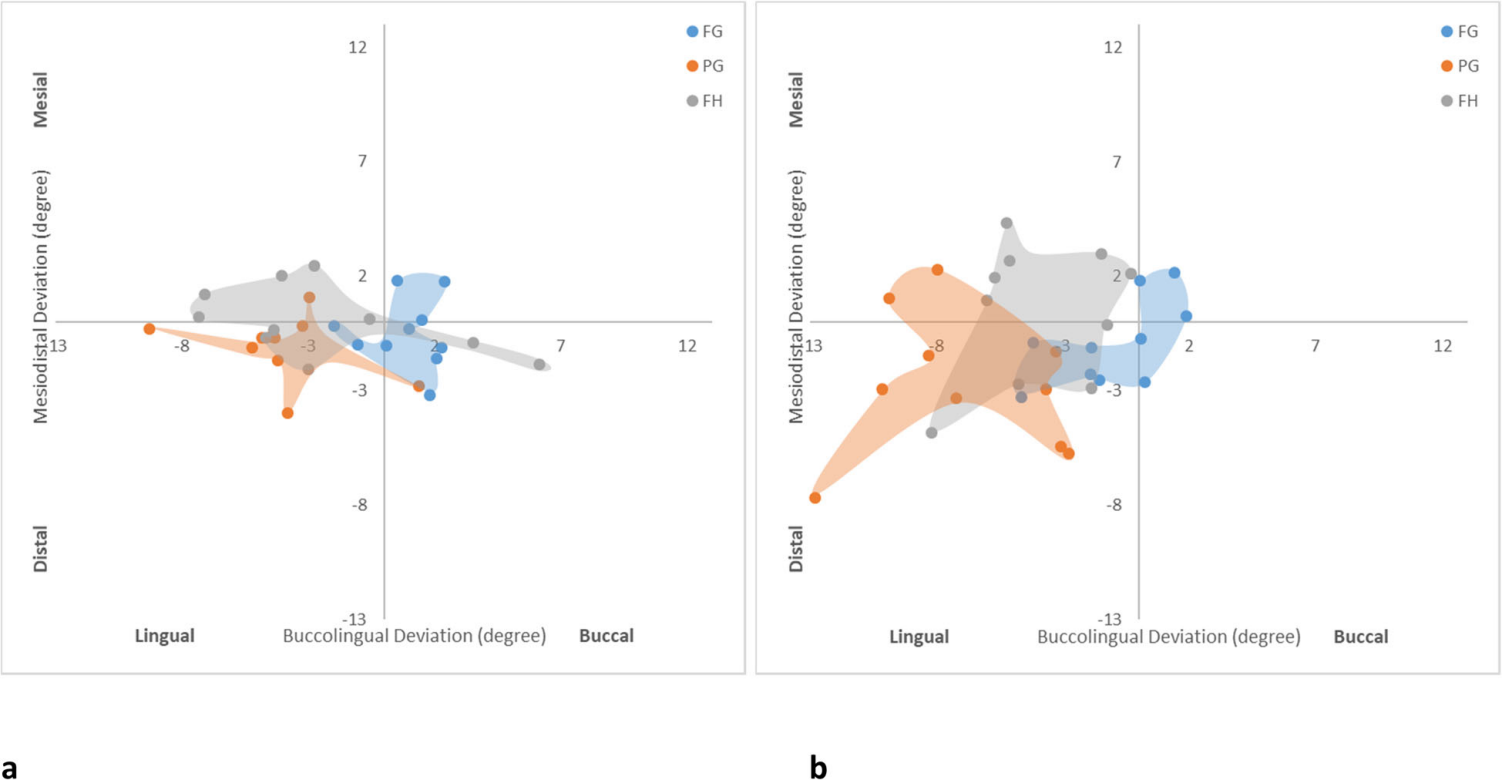
Scatter diagrams illustrating the distribution of angle deviation of each protocol. **a** Anterior implants. **b** Posterior implants

In relation to the maximum angle deviation (Fig. 8), the FG protocol had less deviation than the other protocols for anterior (2.42 ± 0.98°) and posterior (2.61 ± 1.23°) implants. The PG (4.65 ± 1.78°) and FH (4.79 ± 2.08°) protocols were similar for anterior implant placement, while the FH protocol seemed more accurate for posterior implants (4.77 ± 2.09°) than the PG protocol (7.79 ± 2.64°). The FG and FH protocols showed similar angle accuracy for anterior and posterior implants. However, the PG protocol showed inferior angle accuracy of posterior implants than anterior implants. Figure 9 indicates that the FG protocol implant angulations were centered to the middle of the graph confirming the least deviation in relation to the other protocols. The PG protocol showed a tendency to be skewed to the lingual aspect for anterior and posterior implants. The lingual tilting was more noticeable for the posterior implants. In addition, the posterior PG protocol implants were associated with distal deviation. Similarly, the FH protocol showed more lingual tilting.

## Discussion

The overall outcome of this study indicates the superiority of the FG protocol in comparison to PG and FH protocols for placing single implants. With the exception of vertical deviation, this was obvious for horizontal neck, horizontal apex, and angle deviations that were closer to the planned implant for the FG protocol than the other protocols. In addition, this superiority was shown for anterior and posterior implants. Such observations confirm the advantage of the FG protocol in controlling all the steps of osteotomy and implant placement [7, 9, 15, 16]. On the contrary, the inaccuracies of the PG and FH protocols were generally similar and tended to be approximately double the inaccuracies of the FG protocol. Thus, the hypothesis that the accuracies of all the protocols were similar was rejected. In the present study, different software programs were needed to design guides for the FG and PG protocols. While this may have influenced the outcome, the differences between the FG and PG protocols seem to be related to the variations in drilling and implant placement. In addition, since the anterior and posterior implants of the FG protocol had similar accuracy, the hypothesis that there is no influence of the location of the implant on the accuracy of implant placement was accepted. However, this hypothesis cannot be accepted for the PG and FH protocols as the anterior implants were generally more accurate than the posterior implants. Therefore, as per earlier studies, inexperienced clinicians may benefit from FG implant placement [12–14]. For example, Rungcharassaeng et al. found that the FG protocol reduced differences between experienced and inexperienced operators for placing single posterior implants [12]. Likewise, Park et al. and Marheineke et al. found no difference between experienced and inexperienced operators when FG protocol guides were used, while the differences became obvious when implants were placed without surgical guides [13, 14]. According to Schulz et al., in the hand of final-year dental students, FG implant placement was more accurate than PG implant placement [15].

The superior accuracy and the less variation of the FG protocol is most likely related to the control of all the drilling steps and the implant placement via sequential use of precision sleeves. This eliminated the manual orientation and handling of the drills at any stage of drilling or implant placement. In accordance with these observations, Noharet et al. reported a better accuracy of the FG protocol compared with the conventional surgical guide [5]. Likewise, Vermeulen found the FG protocol to be more accurate than the FH protocol [19]. Further, several clinical studies reported that the PG protocol is associated with approximately double the errors of the FG protocol [16, 20, 21]. On the contrary, the PG protocol inaccuracy seemed comparable to the FH protocol, which could be due to the execution of most of the drilling steps and implant placement without the use of guides, leading to inevitable deviation of the drills and implant placement. This is further accentuated in the hands of inexperienced operators who may not precisely control the subsequent drilling steps [14, 15]. While the actual difference between the FG and PG protocols in all the variables is minimal, and still within the recommended safety zone of 2 mm [17], it can still be of clinical significance in cases where the available bone is limited, surgical site is compromised, and the implant is in close proximity to natural teeth and vital anatomical structures [17]. Further, clinically, this will be accentuated with the self-taping abilities of implant threads and inhomogeneity of natural bone that can lead to more implant deviations [20, 21]. Thus, where great accuracy is desirable, the clinician should aim to complete all the drilling procedures and implant placement through the guide [7].

The observed accuracy of the FG protocol (approximate vertical deviation = 0.4 mm, neck deviation = 0.5 mm, apex deviation = 0.7 mm, and angle deviation = 2.5^o^) confirms earlier studies [7, 17, 18] that reported neck deviation in the range of 0.4–0.9 mm [5, 19, 22], apex deviation of 0.5–1.2 mm [5, 19] and angle deviation of 0.3–4.0° [5, 21, 22]. Thus, despite the technological advancement, the FG protocol is still prone to error [4, 7, 9, 12, 18] that is an accumulation of deviations introduced from every step of the planning, guide fabrication, and implant placement procedures [17]. For example, the planning process involves scanning and segmentation of the oral and vital tissues, and any deficiency of the resolution will influence the accuracy of the virtually designed guides [23, 24]. Guides are produced from 3D printing or milling, and both fabrication techniques are susceptible to surface and dimensional errors [6, 25] that may affect the intraoral fit and sleeve orientation. A study that specifically evaluated the errors in the production of guides found that the sleeve centers deviated in the range of 0.07 mm to 0.38 mm, and the angle deviated in the range of 0.4°–3.3° [26]. Nevertheless, the greatest errors seem to occur during the surgical procedure. For example, improper seating of the guide and the deformation of the guide inside the mouth [9, 27]. The deformation of the guide from the present study and from previous studies seem to be more prominent on the buccolingual direction [4, 12, 21, 22], which does not have a rigid structure such as the teeth at the mesiodistal direction. The mechanical tolerance between the drills and the interchangeable sleeves can further contribute to implant deviation [23, 28, 29]. A recent study reported that the length of the sleeve and the drilling distance influenced the accuracy of guided surgery [18]. Further, the presence of debris within the osteotomy can prevent complete seating of the implant [14], which was observed in our study and another study, where the FG protocol implants were more coronal than the planned implants [12]. In clinical situations, more errors are anticipated from CBCT and 3D segmentation of the hard tissues prior to virtual implant planning [23, 24] and patient-related factors such as movement, limited visibility due to the presence of blood, and limited visual access [7, 8].

For the majority of the evaluated variables, there was a tendency for the posterior implants to suffer from more deviation than anterior implants. This is in accordance with several published reports [5, 21, 22]. Interestingly, implants placed by the FG protocol seemed to be less vulnerable to inaccuracy by changing the implant sites, while the PG and FH protocols showed more horizontal and angle deviations for the posterior implants than anterior implants. The inferior outcome of the posterior implants can be due to the limited access, inferior visualization, additional drilling step for wider implants, and more difficult drill orientation for the PG and FH protocols. This also discloses an additional advantage of the FG protocol in being less susceptible to error by altering the implant surgical site, which increases the security during surgery [5, 21, 22].

In accordance with earlier studies, even for the FG protocol, a safety zone is needed and recommended to be in the range of 1–2 mm horizontally and vertically [5, 7, 8, 17], and up to an angle of 5° [7]. While it is tempting to propose a safety zone of 1 mm horizontal and 0.5 mm vertical deviations for the FG protocol as shown by the present study, more errors are expected clinically. Although this study aimed to simulate a clinical set-up, it has limitations that mandate caution while interpreting the results. For example, the models were produced from resin, which does not represent the structure and consistency of natural bone, and may contribute to the greater implant accuracy reported in this study. According to a recent systematic review, implant accuracy was lower in clinical and cadaver studies compared to laboratory studies [7]. The manikin heads with ideal mouth openings do not have natural limiting factors such as blood and saliva and patient movement, limited mouth opening, and restricted interarch clearance. These clinical limitations will interfere with the seating of the guides and orientation of the drills. The FG protocol may even be more influenced especially for posterior implants where the access is limited, that may mandate using the FG protocol guide according to PG protocol. As a result, several authors clearly stated that the use of digital technology does not eliminate the necessity of surgical experience and skills, and the clinicians should be comfortable shifting to conventional implant surgery in case of clinical complications [9, 11, 17]. Due to greater observed error for the PG protocol, it requires a greater safety zone during the planning and the clinician should be prepared to review the osteotomy during the different stages of implant surgery. While superiority of the FG protocol in the range of 0.5 mm–1.0 mm was observed, the deviations of the FG and PG protocols are clinically tolerable, and the differences between them may not be of clinical significance. Further, there is no clinical evidence of difference in implant survival and marginal bone loss of implants inserted conventionally and by the FG protocol [11]. Thus, clinical studies are needed to validate the actual benefit of the FG protocol to justify its routine use for the different clinical presentations [11]. Specifically, if the FG protocol will allow for clinically more esthetic implant restorations, a superior long-term outcome, better soft tissue management, cost-effectiveness, and patient-centered outcome [11]. In addition, the incidence of complications with the FG protocol such as guide misfit, fracture, limited drilling cooling, and lack of implant primary stability [17] should be determined. It is also necessary to emphasize that the results of this study are applicable for single implant placements, and different results may be observed for larger edentulous or longer span areas [4, 17]. This is important as the presence of well aligned teeth and a wide alveolar ridge can be used to guide implant placement to an acceptable orientation, which may explain the general similarity between the PG and FH protocols. Once the presentation becomes more complex, involving more than one implant, the FH implant placement will become more challenging [4, 20].

## Conclusions

Within the limitations of the present study, it can be hypothesized that apart from vertical deviation, the FG protocol is more accurate than the PG and FH protocols for all the evaluated variables in the hands of inexperienced clinicians. The PG and FH protocols were generally similar. The FG protocol did not seem to be influenced by the position of the placed implants, while the PG and FH protocols showed inferior outcomes for the posterior implants in comparison to the anterior implants. The FG protocol of this study indicated that implant position errors were 1 mm horizontally, 0.5 mm vertically, and 2.5° angulation error. This fit within most recommendations that a 2-mm safety zone has to be considered for the FG protocol.

## Data Availability

The datasets used and/or analyzed during the current study are available from the corresponding author on reasonable request.

## Abbreviations

3D: Three-dimensional
CAD/CAM: Computer-aided design/computer-aided manufacturing
CBCT: Cone beam computed tomography
DICOM: Digital Imaging and Communications in Medicine
FG: Fully guided
FH: Freehand
PG: Pilot-guided
sCAIP: Static computer-assisted implant placement
STL: Surface tessellation language
